# Clinical features and genetic analysis of A20 haploinsufficiency

**DOI:** 10.1186/s13023-025-04004-8

**Published:** 2025-08-26

**Authors:** Fumin Xue, Chao An, Zhi Lei, Shijie Dong, Yaqiong Guo, Jiangshan Hou, Jing Yu, Yuesheng Wang

**Affiliations:** 1 Henan Key Laboratory of Children’s Genetics and Metabolic Diseases, Children’s Hospital Affiliated to Zhengzhou University, Zhengzhou, 450018 Henan China; 2 Department of Gastroenterology, Children’s Hospital Affiliated to Zhengzhou University, Zhengzhou, 450018 Henan China; 3 Key Laboratory of Clinical Laboratory Diagnostics, The Second Hospital Affiliated to Zhengzhou University, Zhengzhou, 450018 Henan China; 4 Department of Radiology, Children’s Hospital Affiliated to Zhengzhou University, Zhengzhou, 450018 Henan China

**Keywords:** A20 haploinsufficiency, Monogenic autoinflammatory disease, Genetic mutation, Treatment outcome

## Abstract

**Objective:**

To described clinical and genetic characteristics of 4 patients presenting A20 haploinsufficiency (HA20) treated at Children’s hospital affiliated to Zhengzhou university from 2015 to 2024.

**Methods:**

A retrospective analysis was conducted on the clinical data, genetic testing results, and treatment outcomes of four children with HA20 treated at the Children’s hospital affiliated to Zhengzhou university from 2015 to 2024.

**Results:**

All four patients developed symptoms before the age of 1 year, presenting with recurrent fever and abdominal pain with diarrhea. Most common characteristics were hematochezia, bipolar aphthosis, arthritis, skin eruption in 50% of patients. Lab tests revealed elevated inflammatory markers; all patients had anemia. Imaging showed intestinal mucosal edema, hip/knee joint effusions, and lymphadenopathy in one case. Endoscopy revealed gastrointestinal aphthosis in 100% of cases. Genetic testing identified *TNFAIP3* mutations in all four patients, including one novel whole-gene deletion (6q23.3chr6:136700000–138880000), 2 novel pathogenic mutations (c.866delA, c.1243_1247del), and one previously reported mutation (c.133C > T). Treatment included exclusive enteral nutrition (EEN) and thalidomide for all patients. One patient was switched to infliximab (IFX) combined with azathioprine due to gastrointestinal side effects, and one patient received methylprednisolone during acute phase. Follow-up for 4–10 years showed that 1 patient had improved symptoms with IFX and azathioprine but still had intermittent fever and perianal aphthosis; While one patient demonstrated poor response to EEN-thalidomide therapy requiring regimen change, the other responded well. One patient exhibited normalized gastrointestinal function following EEN-thalidomide therapy, yet still required repeated hospitalizations for recurrent infections with progressively prolonged inter-episode intervals.

**Conclusion:**

HA20 has strong clinical heterogeneity, and genetic testing is crucial for diagnosis and guiding treatment. Early diagnosis and individualized treatment can improve prognosis.

## Introduction

A20, a tumor necrosis factor α-induced protein 3 *(TNFAIP3)* -encoded protein characterized by an N-terminal ovarian tumor (OTU) deubiquitinase domain and seven C-terminal zinc finger (ZnF) domains, acts as a potent negative regulator of the NF-κB pathway[1]. A20 haploinsufficiency (HA20) is an autosomal dominant genetic disorder caused by mutations in the *TNFAIP3* gene and is classified as a monogenic autoinflammatory disease (mAIDS). It typically manifests in infancy, characterized by multi-organ inflammatory and autoimmune reactions [1]. The clinical features of HA20 are highly heterogeneous [2, 3], with common presentations resembling Behçet’s disease (BD), including recurrent fever, bipolar aphthosis, gastrointestinal aphthosis, skin eruption and musculoskeletal damage. The correlation between different *TNFAIP3* gene mutation sites and the heterogeneity of clinical symptoms is significant; therefore, identifying the specific *TNFAIP3* mutation is crucial for diagnosis, treatment selection, and prognosis assessment.

This study retrospectively reviewed the clinical and genetic characteristics of 4 patients presenting HA20 treated at the Children’s Hospital Affiliated to Zhengzhou university from 2015 to 2024 to enhance the understanding of this disease.

## Materials and methods

### Study design and patient selection

This retrospective cohort study included four children diagnosed with HA20 at the Children’s Hospital Affiliated to Zhengzhou university between January 2015 and December 2024. Inclusion criteria required confirmation of *TNFAIP3* pathogenic variants or deletions via whole-exome sequencing (WES). Patients with BD-like symptoms lacking genetic confirmation or ambiguous diagnoses were excluded.

### Data collection

Clinical data were extracted from electronic medical records, including:

1. Demographics: age, sex, family history, ethnicity.
2. Clinical Features: recurrent fever, gastrointestinal symptoms (abdominal pain, diarrhea, hematochezia), mucocutaneous aphthosis, arthritis, and extraintestinal manifestations.
3. Laboratory Investigations: complete blood count, inflammatory markers (ESR, CRP, IL-6), liver/kidney function tests, autoantibody panels (19 items), and pathogen screening.
4. Imaging: abdominal CT, joint MRI, and gastrointestinal endoscopy.
5. Genetic diagnosis results.
6. Treatment and Outcomes: therapies administered, clinical response, and complications.

### Genetic analysis

Peripheral blood samples (2 mL) from patients and first-degree relatives underwent WES using Illumina platforms. Samples were sent to Beijing GrandOmics for genomic DNA extraction. WES libraries were prepared using the xGen® Exome Research Panel v1.0 (Integrated DNA Technologies, USA) for target capture. Libraries underwent sequencing on the Illumina NovaSeq 6000 platform. Total coverage was over 99%, the average sequencing depth was over 200 × , and the coverage at ≥ 20 × depth was over 98%. Variants were then filtered for potential relevance to the proband’s phenotype. Identified variants were interrogated against major databases (HGMD, ClinVar, dbSNP, ExAC, OMIM, PubMed) to assess their known associations and existing literature. Pathogenicity classification was performed according to the standards and guidelines established by the American College of Medical Genetics and Genomics (ACMG)[4]. Candidate variants underwent validation via Sanger sequencing to confirm their genetic origin.

### Statistical and ethical considerations

**Fig. 1:**
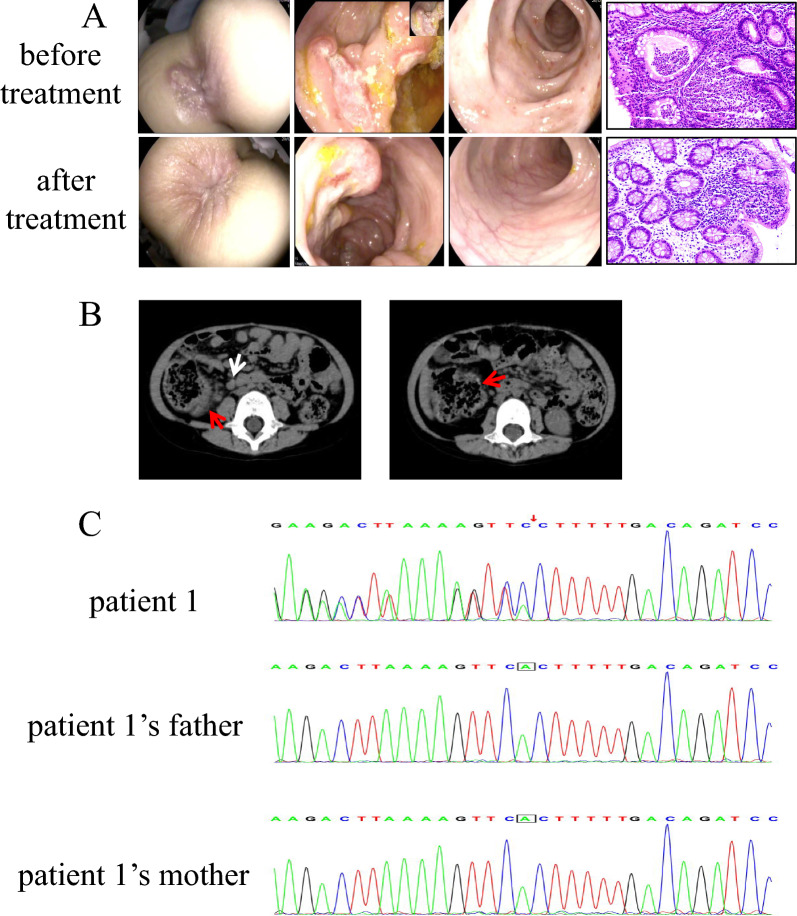
**A** Pre- and post-treatment colonoscopy findings and histopathological images (20 ×) in Patient 1; **B** Abdominal CT imaging of Patient 1. White arrow: Enlarged abdominal lymph nodes; Red arrow: Intestinal edema with luminal dilation; **C** WES for Patient 1 and her parents. A heterozygous *TNFAIP3* mutation (c.866delA) was identified in the proband, while both parents exhibited wild-type genotypes

**Fig. 2:**
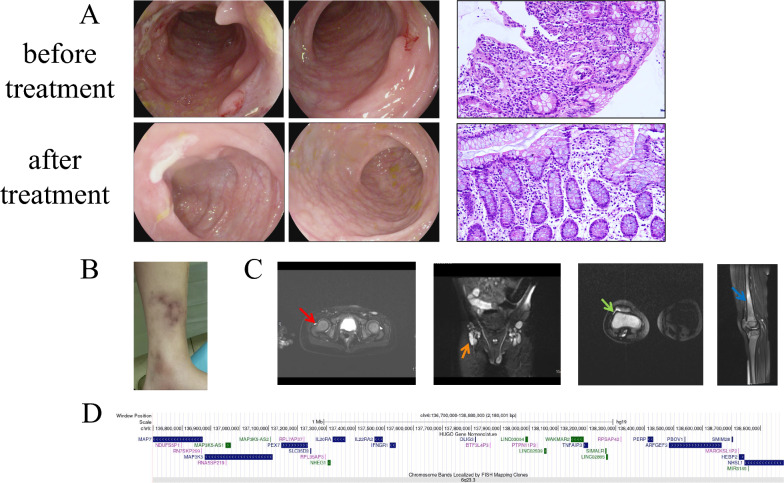
**A** Endoscopic progression and histopathological images (20 ×) in Patient 2 before and after therapy; **B** Nodular erythematous changes in the left lower extremity; **C** MRI evaluation of hip and knee joints. Hip Joints (both sides): Fluid accumulation in joint spaces (red arrow); Inflamed nodules (orange arrow); Right Knee Joint: Abnormal fluid collection within the joint (green arrow); Bone tissue inflammation in femur and lower leg bones (blue arrow); **D** A large deletion in chromosome 6 and OMIM genes encompassing the 6q23.3 region

**Fig. 3:**
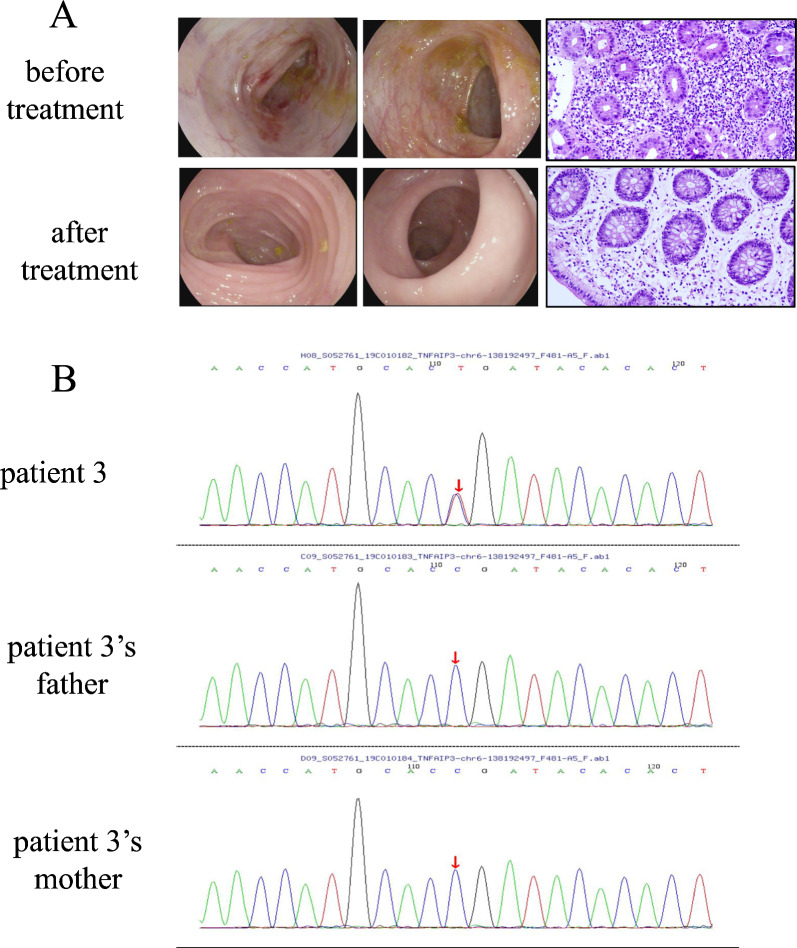
**A** Serial colonoscopy images and histopathological images (20 ×) documenting therapeutic response in Patient 3; **B** Familial WES result. Patient 3 carries a heterozygous *TNFAIP3* variant (c.133C > T), absent in wild-type parents

Descriptive statistics summarized clinical and laboratory data. The study was approved by the hospital’s ethics committee, with informed consent obtained from patients’ parents (Figs. 1, 2, 3).

## Results

### Clinical and demographic characteristics

**Fig. 4:**
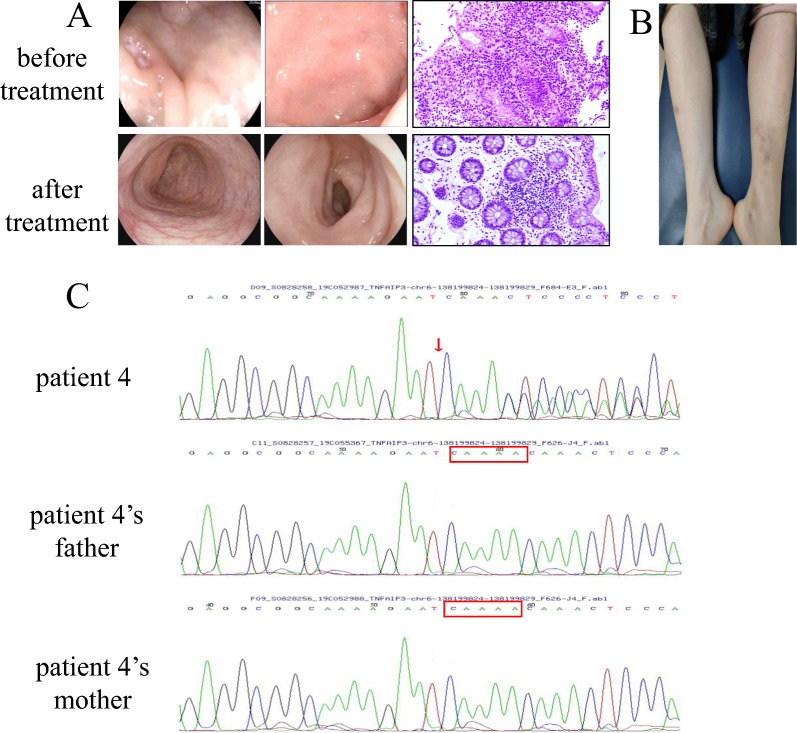
**A** Colonoscopic evolution and histopathological images (20 ×) in Patient 4 during treatment. **B** Multiple dark red maculopapular lesions on bilateral tibial skin. **C** WES reveals a de novo frameshift mutation in *TNFAIP3* (c.1243_1247del) in Patient 4, with both parents showing wild-type sequences

Four pediatric patients (2 males, 2 females) with HA20 were included, all exhibiting symptom onset before 12 months of age. Common manifestations included recurrent fever (4/4), abdominal pain with diarrhea (4/4), hematochezia (2/4), and mucocutaneous aphthosis(2/4). While 2 patients had skin eruption(2/4, patient 2 and patient 4). Patient 2: Erythema nodosum presenting as tender, 0.5–2.0 cm violaceous ubcutaneous nodules with coalescent plaques and ecchymotic borders; no ulceration (Fig. 2B). Patient 4: Dark red maculopapular lesions, slightly raised with erythematous halos; no nodules, ulceration, or tenderness (Fig. 4B). And both patients exhibited concurrent extraintestinal manifestations (2/4). Patient 2 developed arthritis, while Patient 4 presented with transient liver injury. No ocular or neurological involvement was observed (0/4) (Table 1).

**Table 1:** The clinical presentations of the 4 HA20 patients

	Patient 1	Patient 2	Patient 3	Patient 4
Age of onset	11 months	2 months	10 months	6 months
Gender	Female	Male	Female	Male
Ethnicity	Han	Han	Han	Han
Fever	+	+	+	+
Abdominal pain/diarrhoea	+	+	+	+
Hematochezia	+	+	−	−
Oral aphthosis	+	+	−	−
Genital aphthosis	+	+	−	−
Arthritis	−	+	−	−
Oculopathy	−	−	−	−
Skin eruption	−	Erythema nodosum	−	Dark red maculopapular eruptions
Hepatic injury	−	−	−	+
CNS symptoms	−	−	−	−
Treatment	EEN, thalidomide(Discontinued due to gastrointestinal side effects), IFX	EEN, GC (Taper off gradually), thalidomide	EEN, thalidomide	EEN, thalidomide

“ − ”means “Negative”;

“ + ” means “Positive”

CNS: Central Nervous System, EEN: exclusive enteral nutrition, IFX: infliximab, GC:Methylprednisolone

### Laboratory and imaging findings

At initial presentation, all patients showed leukocytosis (WBC: 13.41–28.2 × 10^9/L), thrombocytosis (PLT: 359–594 × 10^9/L), anemia (HGB: 62–110 g/L), and elevated inflammatory markers (CRP: 100–166.68 mg/L; ESR: 19–112 mm/h; IL-6: 32.51–58.4 pg/mL). Liver enzymes (ALT/AST) were elevated only in Patient 4 (ALT: 165.6 U/L; AST: 172.9 U/L). Autoantibody panels, rheumatoid factor, skin prick test and pathogen screening were negative. (Table 2).

**Table 2:** Laboratory and imaging collection of 4 HA20 patients

	Patient 1	Patient 2	Patient 3	Patient 4
WBC (3.69–9.16 × 10^9^/L)	**16.78**	**16.4**	**13.41**	**28.2**
NE (50–70%)	63.74	61.7	69	**39.7**
HGB (110–150 g/L)	**82**	**62**	**94.2**	110
PLT (100–300 × 10^9^/L)	**359**	**594**	**454**	**419**
ESR (0–20 mm/h)	**97**	**112**	19	**33**
CRP (0–10 mg/L)	**166.68**	**100**	**108.54**	**163.86**
ALT (0–40 U/L)	20.2	21.4	18.3	**165.6**
AST (0–40 U/L)	32.8	28.6	34.3	**172.9**
ALB (39–54 g/L)	43	**32.6**	**28.1**	**29.9**
CREA (13–33 µmol/L)	24.1	18	20.3	23.6
LDH (120–246 U/L)	235.4	**252.7**	**274.1**	**306**
IL-6 (0–7 pg/mL)	**36**	**58.4**	**32.51**	**36.92**
Autoantibody panel (19 items)	−	−	−	−
Lymphocyte subset analysis	**CD8 + T cells increased (11–41%); B cells decreased(6–25%)**	**CD4 + T cells decreased(31–60%)**	Negative	Negative
Humoral immunity (g/L)	**C4 (0.2–0.4 g/L)and IgA (0.58–2.91 g/Lelevated**	**C3(0.8–1.6 g/L) and C4 (0.2–0.4 g/L)elevated**	**C4 (0.2–0.4 g/L) elevated**	Negative
RF (0–20 RU/ml)	**RF-IgA elevated**	**RF-IgA elevated**	**Negative**	**RF-IgA elevated**
CCP (0–5 RU/ml)	3.76	1.63	2.44	2.13
ASO (0–200 IU/mL)	12.6	25.3	19.68	18.38
Blood and stool pathogen testing	−	−	−	−
Skin prick test	−	−	−	−
Ultrasound	**Edema of the intestinal mucosa**	**Effusion in the right knee with synovial thickening**	**Edema of the intestinal mucosa**	**Edema of the intestinal mucosa, with enlargement of abdominal lymph nodes**
Electromyography	/	−	/	/
Abdominal CT	**Bowel dilation with lymph node enlargement**	/	/	/
Abdominal MRI	/	**Right knee effusion, synovial thickening, and bone marrow hyperintensity**	/	/
Brain MRI	/	−	/	/
bone marrow aspiration	/	−	/	/
gastroscopy	−	**Gastric ulcer**	−	−
colonoscopy	**Deep and large ulcers in the entire colon**	**Ulceration of the entire colon**	**Mucosal erosion below the transverse colon**	**Multiple ulcers in the colonic mucosa**

WBC: white blood cells, NE: neutrophil count, HGB: hemoglobin, PLT: platelet, ESR: erythrocyte sedimentation rate, CRP: C-reactive Protein, ALT: alanine aminotransferase, AST: aspartate aminotransferase, ALB: albumin, CREA: creatinine, LDH: lactate Dehydrogenase, IL: interleukin, RF: rheumatoid factor, CCP: cyclic citrullinated peptide, ASO: antistreptolysin O, CT: computed tomography, MRI: magnetic resonance imaging, Abnormal results are displayed in large, bold type

“ − ”means “Normal”; “/” means “patient didn’t conduct the test or scan”;

Imaging revealed intestinal mucosal edema in all patients (4/4). Patient 2 displayed right knee effusion, synovial thickening, and bone marrow hyperintensity on MRI, suggestive of active hematopoiesis (Table 2, Fig. 2C). Further bone marrow aspiration was normal. Electromyography, brain MRI, and lumbosacral MRI showed no abnormalities (Table 2). Gastrointestinal endoscopy identified extensive colonic aphthosis in Patients 1 and 2, while Patients 3 and 4 exhibited localized mucosal erosions or aphthosis (Figs. 1A, 2A, 3A, 4A,). Gastrointestinal endoscopy revealed gastric aphthosis in Patient 2.

### Genetic variants

Whole-exome sequencing identified heterozygous *TNFAIP3* (NM_006290.4) mutations or deletion in all patients (Table 3, Figs. 1C, 2D, 3B, 4C).

**Table 3:** Gene information of 4 patients with *TNFAIP3* mutation

	Patient 1	Patient 2	Patient 3	Patient 4
Genomic location (hg19)	chr6:138198273	6q23.3 chr6:136700000–138880000	chr6:138192497	chr6:138199829–138199833
Exons	6	–	2	7
Nucleotide change	c.866delA	–	c.133C > T	c.1247_1251del
Protein change	p.(His289Profs*3)	–	p.(Arg45Ter)	p.(Asn416Thrfs*11)
Type of variant	Frameshift	Deletion	Nonsense	Frameshift
Zygosity	Heterozygous	Heterozygous	Heterozygous	Heterozygous
Inheritance	de novo	de novo	de novo	de novo
Domain	OTU	–	OTU	ZnF1
MAF	–	–	–	0.00001
CADD	Deleterious (34.0)	–	Deleterious (46.0)	Deleterious (25.2)
Mutation detection strategy	WES	WES + CNV-seq	WES	WES

*TNFAIP3* transcript: NM_006290.4, MAF: Minor Allele Frequency, CADD: Combined Annotation Dependent Depletion, WES: Whole-exome sequencing, CNV-seq: Copy number variation sequencing

Patient 2 harbored a complete *TNFAIP3* deletion (6q23.3chr6:136700000–138880000), the deletion leads to A20 haploinsufficiency. While Patients 1, 3, and 4 carried novel frameshift or nonsense mutations:

Patient 1: c.866delA (p.His289Profs*3), a frameshift variant in the OTU domain.

Patient 3: c.133C > T (p.Arg45Ter), a nonsense mutation truncating the OTU domain. This variants has been previously reported [5, 6].

Patient 4: c.1243_1247del (p.Asn416Thrfs*11), a frameshift mutation occurring between the ZnF1 and ZnF2 domains.

**Fig. 5:**
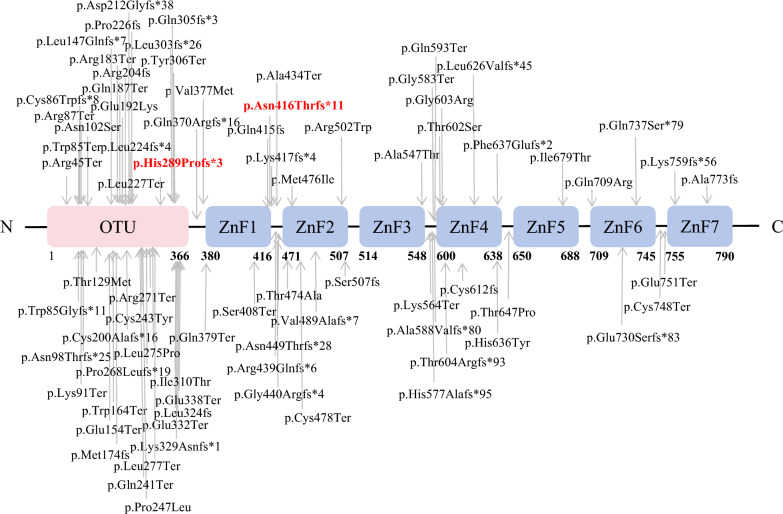
Schematic representation of reported variants and our study (red color: new variants) in *TNFAIP3* gene

Variants c.866delA (PVS1_Very strong + PS2_Moderate + PM2_Supporting), c.133C > T (PVS1_Very strong + PS2_Moderate + PM2_Supporting) and c.1243_1247del (PVS1_Very strong + PS2_Moderate + PM2_Supporting) were classified as pathogenic per ACMG criteria and confirmed as de novo via parental testing (Fig. 5).

### Treatment outcomes

All patients received EEN combined with thalidomide (1.5 mg/kg/day).

Patient 1 required IFX (5 mg/kg) and azathioprine due to thalidomide intolerance, achieving partial symptom control.

Patient 2 received adjunctive methylprednisolone during flares but exhibited persistent mucosal aphthosis despite therapy.

Patient 3 attained complete clinical and endoscopic remission after 9 years of EEN/thalidomide.

Patient 4 experienced recurrent infections but showed gradual improvement in liver function and gastrointestinal symptoms.

Over a 4–10-year follow-up, disease activity varied: Patients 1 and 2 had residual symptoms, while Patients 3 achieved sustained remission. Patient 4 demonstrated sustained remission of gastrointestinal manifestations, with no recurrence observed during the follow-up period. (Table 1).

## Discussion

The A20 acts as a critical negative regulator of the NF-κB signaling pathway by suppressing inflammatory cascades through its dual enzymatic domains(OTU and ZnFs) [7, 10, 31]. Beyond NF-κB, emerging evidence highlights its regulatory roles in the JNK and JAK-STAT pathways, underscoring its broad influence on immune homeostasis [8]. Expressed ubiquitously in immune and epithelial cells, A20 modulates diverse processes, including apoptosis, inflammation, and oncogenesis [9]. Structural analysis reveals that the OTU domain deubiquitinates key mediators of the IKK complex, dampening NF-κB activation, while the ZnF domain facilitates RIPK1 ubiquitination and degradation, further curbing inflammatory signaling [8, 11]. Loss of A20 function disrupts this regulatory balance, leading to unchecked NF-κB activation and predisposing individuals to autoimmune and autoinflammatory disorders [12, 13].

Patients with HA20 demonstrate a broad clinical spectrum characterized by multisystem involvement [20]. The predominant manifestations include recurrent oral aphthosis (present in 68% of cases), genital aphthosis (37%), periodic fever patterns (48%), cutaneous eruptions (41%), and gastrointestinal pathology manifesting as abdominal pain, diarrhea, or hematochezia (39%) [14, 15, 20]. Lymphadenopathy and recurrent respiratory infections are frequently observed comorbidities. Notably, approximately 30% of patients develop autoimmune sequelae ranging from Hashimoto’s thyroiditis to systemic lupus erythematosus [16], and rheumatological complications such as arthritis [17]. Less common but clinically significant associations include interstitial pneumonia, neuroinflammatory manifestations, and hematological malignancies like Hodgkin’s lymphoma [14, 15, 17].

Geographical variation in phenotypic expression has been well-documented. East Asian cohorts predominantly exhibit periodic fever syndromes of unknown etiology (70.4% vs. 37.3% in non-Asian populations, *P* < 0.001), whereas Mediterranean and European populations more frequently present with Behçet’s disease-like symptomatology [14]. This regional disparity extends to autoimmune manifestations, with East Asian patients demonstrating lower incidence rates of autoimmune diseases (29.2% vs. 58.8%, *P* < 0.01) compared to other ethnic groups.

The molecular pathogenesis of HA20 centers on *TNFAIP3* mutations disrupting A20’s dual regulatory domains [18]. Since the initial 2016 characterization, 75 genetic variations at the *TNFAIP3* locus have been cataloged [19–22], and 12 cases of chromosome 6q23 segmental deletions causing complete A20 deficiency [23, 24]. These large deletions (median size 3.35 Mb) typically abrogate both OTU and ZnF domains, resulting in constitutive NF-κB activation through loss of deubiquitinase and ubiquitin-binding functions. Clinically, complete A20 deficiency correlates with early disease onset (median age 4 months), severe multiorgan involvement, and neurodevelopmental abnormalities (4.2% incidence) [24–26]. Our Patient 2 exemplifies this severe phenotype, presenting at 2 months with pan-gastrointestinal ulceration and progressing to joint destruction, highlighting the prognostic challenges in this subgroup requiring vigilant neurological monitoring. Patient 2 harbors a 2.18 Mb heterozygous deletion on chromosome 6 (6q23.3), resulting in the loss of 13 protein-coding genes, notably *TNFAIP3, PEX7, IL20RA,* and *IL22RA2*. The *TNFAIP3* deletion, causative of HA20, underlies the core Behçet’s-like phenotype characterized by fever, abdominal pain, diarrhea, hematochezia, skin eruption, Oral aphthosis and genital aphthosis, intestinal ulcers, and arthritis. The observed arthritis may potentially relate to *PEX7* deletion [27], while deletions in *IL20RA* and *IL22RA2* might exacerbate inflammatory dysregulation or skin eruption [28].

Mutation-specific genotype–phenotype correlations continue to evolve. Frameshift/nonsense mutations predispose to immune dysregulation with recurrent infections and cytopenias, while missense variants associate with cutaneous vasculitis [18, 24]. Domain-specific analyses reveal OTU domain mutations preferentially link to Behçet’s-like phenotypes, whereas combined OTU + ZnF disruptions correlate with musculoskeletal complications, suggesting that the ZnF domain may play an important role in the pathogenesis of musculoskeletal diseases in HA20 patients [29]. ZnF7 domains deletion have been reported in mouse to result in a spontaneous inflammatory disease, but not ZnF4 deletion [30]. However, recent meta-analyses challenge these associations, emphasizing the need for larger cohort studies to resolve current controversies in phenotypic stratification [12, 24].

In this study, Patients 1 and 4 both exhibited frameshift mutations at previously unreported sites. These distinct mutations correlated with differing clinical presentations. Patient 1 carried a *TNFAIP3* mutation (c.866delA), leading to p.His289Profs*3. This highly pathogenic frameshift mutation disrupts part of the OTU domain and causes complete loss of all zinc finger domains (ZnFs). The resulting severe impairment of A20 function manifested as significant gastrointestinal symptoms and recurrent infections, with suboptimal therapeutic response. Patient 4, however, harbored a *TNFAIP3* variant (c.1243_1247del; p.Asn416Thrfs*11). This mutation preserved the OTU and ZnF1, resulting only in the loss of ZnF2-ZnF7. The clinical presentation featured predominant recurrent infections and milder gastrointestinal symptoms. The observed liver injury and rash support the proposed role of ZnF domains in regulating organ-specific inflammation [24]. These may also suggest an association between OTU domain alterations and Behçet’s-like phenotypes [29]. Patient 3 presented a nonsense mutation (c.133C > T; p.Arg45Ter), causing A20 truncation. This abolished both the OTU domain’s deubiquitinase activity and the ubiquitin ligase activity of all zinc fingers. Remarkably, after nine years of follow-up, this patient has achieved complete clinical and endoscopic remission. These findings show both consistency and contradictions with Chen et al.’s research [29], underscoring the disease’s complexity and the critical need to expand the catalog of documented *TNFAIP3* variants. This study describes four *TNFAIP3* variants, three of which are novel (not previously reported). Our primary contribution lies in enriching the existing repository of *TNFAIP3* variants. At present, definitive genotype–phenotype correlations cannot be established based on this cohort.

The establishment of definitive diagnostic criteria for HA20 remains an unmet clinical need, complicated by its phenotypic heterogeneity and lack of pathognomonic laboratory findings. During acute flares, elevations in ESR, CRP, proinflammatory cytokines (e.g., IL-6), and autoantibodies may occur, though these markers merely reflect systemic inflammation rather than providing diagnostic specificity. In our cohort, all four patients demonstrated anemia (likely secondary to chronic gastrointestinal bleeding) with Patient 2 exhibiting severe anemia requiring hematological intervention. Joint MRI in this patient revealed compensatory erythroid hyperplasia. While immune dysregulation is central to HA20 pathogenesis [31]. Evidenced by T/B lymphopenia in Patients 1 and 2 potentially reflecting chronic inflammation-induced cytotoxicity, these cellular alterations lack diagnostic specificity. Experimental evidence implicates *TNFAIP3* deficiency in immune tolerance breakdown, with dendritic cell-specific knockouts developing SLE-like phenotypes [32] and B cell-specific deletions causing spontaneous autoantibody production [33]. Such mechanisms may explain the observed complement activation (elevated C3/C4) and cytokine-driven T/B cell dysfunction in HA20 patients [31, 34]. Genetic confirmation through whole-exome or genome sequencing has become the diagnostic gold standard. We recommend prompt genetic evaluation for patients presenting with: (1) Early-onset disease (< 12 months); (2) Recurrent fever of unknown origin; (3) Mucocutaneous aphthosis (oral/genital/gastrointestinal); (4) Unexplained multiorgan involvement.

Therapeutic strategies remain empirical, reflecting the disease’s variable clinical trajectories. First-line corticosteroids, though effective for acute control, face adherence challenges due to growth-related complications in pediatric populations. Conventional immunosuppressants (methotrexate, azathioprine) typically require glucocorticoid co-administration for sustained remission [8]. Emerging biologic therapies-including TNF-α inhibitors (infliximab), IL-1/IL-6 pathway antagonists (anakinra, tocilizumab), and JAK inhibitors (tofacitinib)—offer targeted modulation of hyperinflammatory responses, particularly in refractory cases [35]. Critical considerations for treatment personalization include: Developmental stage-adjusted dosing regimens; Predominant pathophysiological drivers (autoinflammatory vs autoimmune dominance); Mutation-specific functional impacts (OTU vs ZnF domain perturbations). In this report, All patients received EEN and thalidomide, a regimen effective in mild-moderate cases. Patient 1’s switch to infliximab after thalidomide intolerance highlights TNF-α’s central role in HA20 pathogenesis, particularly in OTU domain defects. Conversely, Patient 2’s refractory disease may reflect irreversible NF-κB hyperactivation due to complete A20 loss, necessitating combination biologics (e.g., IL-1/IL-6 inhibitors) or JAK inhibitors. The variability in mucosal healing complete in Patient 3 versus persistent aphthosis in Patient 2 suggests that residual A20 function, dictated by mutation type, influences tissue repair capacity.

This study’s small sample size and retrospective design limit generalizability. Longitudinal follow-up (4–10 years) revealed evolving phenotypes, yet longer observation is needed to assess late complications (e.g., malignancy). Furthermore, functional studies are imperative to clarify how specific mutations dysregulate A20’s interactions with RIPK1 or TRAF6. Multicenter cohorts and standardized treatment protocols are essential to validate genotype-driven therapies and establish prognostic biomarkers.

## Conclusion

HA20’s clinical heterogeneity underscores the importance of genetic testing for diagnosis and personalized management. This study reinforces the association between mutation type and disease severity while identifying novel variants. Future research should prioritize molecular mechanistic studies and randomized trials to optimize therapeutic strategies for this evolving disorder.

## Data Availability

All the data and materials are available from the corresponding authors upon request.

## Abbreviations

HA20: A20 haploinsufficiency
EEN: Exclusive enteral nutrition
IFX: Infliximab
TNFAIP3: Tumor necrosis factor α-induced protein 3
OUT: Ovarian tumor
ZnF: Zinc finger
mAIDS: Monogenic autoinflammatory disease
BD: Behçet’s disease
ACMG: Medical Genetics and Genomics
CNS: Central Nervous System
GC: Methylprednisolone
WBC: White Blood Cells
NE: Neutrophil count
HGB: Hemoglobin
PLT: Platelet
ESR: Erythrocyte Sedimentation Rate
CRP: C-reactive Protein
ALT: Alanine Aminotransferase
AST: Aspartate Aminotransferase
ALB: Albumin
CREA: Creatinine
LDH: Lactate Dehydrogenase
IL: Interleukin
RF: Rheumatoid Factor
CCP: Cyclic Citrullinated Peptide
ASO: Antistreptolysin O
CT: Computed Tomography
MRI: Magnetic Resonance Imaging
MAF: Minor Allele Frequency
CADD: Combined Annotation Dependent Depletion
WES: Whole-exome sequencing
CNV-seq: Copy number variation sequencing
